# Hand osteoid osteoma: evaluation of diagnosis and treatment

**DOI:** 10.1186/s40001-019-0361-1

**Published:** 2019-01-21

**Authors:** Ozgur Erdogan, Volkan Gurkan

**Affiliations:** 1 Department of Orthopaedics, Haydarpasa Numune Training and Research Hospital, Health Sciences University, Tibbiye Cd No: 40 Uskudar, Istanbul, Turkey; 2 Department of Orthopaedics, Faculty of Medicine, Bezmialem Vakif University, Vatan Cd, Fatih, 34093 Istanbul, Turkey

**Keywords:** Hand, Metacarpal, Osteoid, Osteoma, Phalangeal

## Abstract

**Background:**

OO (osteoid osteoma) is a common, osteoblastic, benign bone tumor but rarely seen in the hand region. There is still some debate about the diagnosis and treatment of hand OOs. In the present study, we aimed to evaluate the epidemiology, radiologic features, surgical treatment options and functional outcomes.

**Methods:**

Between January 2003 and December 2014, surgically treated and pathologically verified 9 hand OO cases were investigated retrospectively. The preoperative and postoperative clinical outcome scores were calculated using the M2-DASH (Manchester-Modified Disabilities of Arm Shoulder and Hand) Score.

**Results:**

Lesion locations were as follows: middle phalanx in 2/9 (22%) patients (2nd and 4th digit), proximal phalanx in 6/9 (67%) patients (one 4th, two 2nd and three 5th digits) and metacarpal (2nd) in 1/9 (11%) patient. Incidence of nidus formation was 6/9 (67%) on X-ray, 7/9 (78%) on CT imaging and 2/9 (22%) on MR imaging. The mean time to diagnosis was 13.22 ± 5.44 months. Preoperative mean M2-DASH score was 41 ± 6 and postoperative was 7.4 ± 8.6.

**Conclusion:**

Osteoid osteoma is usually seen below 25 years, and rarely found over 40 years of age. There is male dominance with a male to female ratio of 3:1. Delay of diagnosis may be encountered because of many differential diagnoses. When OO is suspected, CT imaging should be taken before the MR imaging. Because of superiority in soft tissue imaging, MR imaging should be an alternative tool in complex cases.

## Background

Osteoid osteoma (OO) is a vascularized, osteogenic, benign bone tumor and was first defined by Heine in 1927 [1] and first described by Jaffe in 1935 [2]. The lesion is characterized as a well-defined lytic area with the vascularized central nidus which is surrounded by sclerosis and cortical thickening in X-ray and computerized tomography (CT) imaging. Magnetic resonance (MR) imaging usually shows an extensive bone marrow and/or soft tissue edema [3–5]. OO is rarely seen in the hand region. Delay of diagnosis can be experienced, because of different clinical, radiological and histological features from the long bone OOs [6, 7]. Further, differential diagnosis and nonspecific findings on radiographs complicate the diagnosis. Most of the papers are case reports, but still, there is a need for case series due to the rarity and difficulties in diagnosing. In the present study, we aimed to evaluate the epidemiology, radiologic features, surgical treatment options and functional outcomes.

## Methods

The study was performed in accordance with the ethical standards of the Declaration of Helsinki. All patients provided informed consent before inclusion in the study, and a local ethics committee approved the study protocol. This study was performed on surgically treated 9 hand OO patients from January 2003 to December 2014. Inclusion criteria were histologically verified metacarpal and phalangeal OO. Patients who had previous percutaneous or surgical treatment and patients with recurrence were excluded from the study. All patients were evaluated regarding swelling, pain, trauma history, night pain, response to pain relievers, duration of complaints and time to diagnosis. All patients were evaluated with X-ray, CT, MR and SPECT-CT imaging preoperatively. The preoperative and postoperative clinical outcome scores were calculated using the M2-DASH (Manchester-Modified Disabilities of Arm Shoulder and Hand) Score [8]. Statistical analysis was performed using SPSS software (IBM, Armonk, NY) using an unpaired Student’s *t* test and the Fisher exact test. Statistical significance level was set at *p* ≤ .05.

## Results

**Fig. 1:**
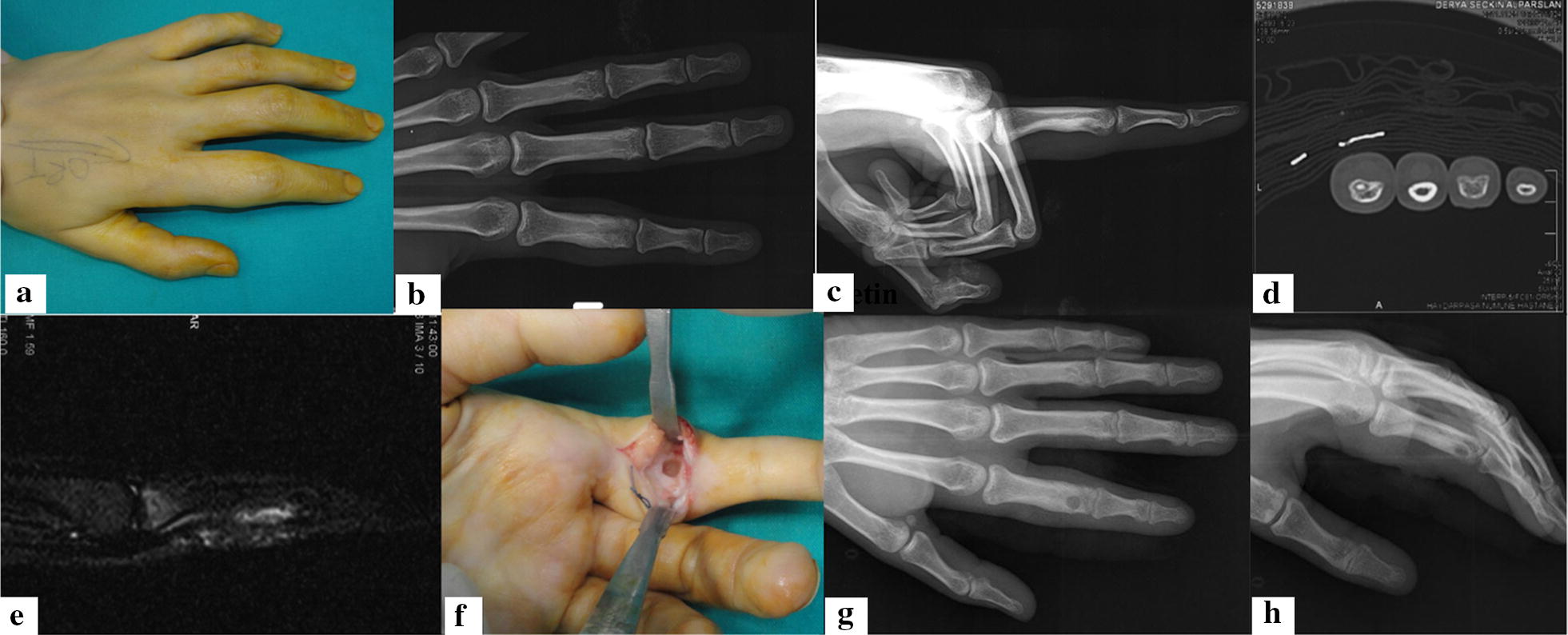
**a** Preoperative anterior–posterior plain graph showing cortical sclerosis. **b** The sole finding was volar cortical thickening in lateral view. **c** CT imaging axial section shows nidus formation clearly. **d** MR imaging sagittal section shows nonspecific edema. **e** Moderate swelling of the second finger was first finding. **f** Perioperative image shows cortical defect after curettage. **g** The postoperative anterior–posterior plain graph shows the cortical defect. **h** Postoperative lateral view shows the curettage area, possibly a little distal from the nidus center, resulting with a residual lesion

Seven (78%) of patients were male, and 2 (22%) were female, and the mean age was 29 ± 7 years. Lesion locations were as follows: proximal phalanx in 6/9 (67%) patients (one 4th, two 2nd and three 5th digits), middle phalanx in 2/9 (22%) patients (2nd and fourth digit), and metacarpal (2nd) in 1/9 (11%) patient. The mean time to diagnosis was 13.22 ± 5.44 months. There were night pain, localized swelling, tenderness and response to pain relievers in all patients. Also, there were slight erythematous changes and local skin temperature increase in 3/9 (33%) patients. Complete blood count, erythrocyte sedimentation rate and C-reactive protein (CRP) levels were normal in all patients. Incidence of nidus formation was 6/9 (67%) on X-ray, 7/9 (78%) on CT imaging and 2/9 (22%) on MR imaging. Cortical thickening rate was 7/9 (78%) on X-ray, 7/9 (78%) on CT imaging and 3/9 (33%) on MR imaging. Non-specific findings were found in 2/9 (22%) cases both on X-ray and CT imaging. Bone and/or soft tissue edema rate was 8/9 (89%) on MR imaging (Table 1). All except one were treated by unroofing and curettage using “Burr-down” method. En bloc resection was performed in one case (11%). Mean M2-DASH score was 41 ± 6 and improved to 7.4 ± 8.6 postoperatively. Complications were seen in 4/9 (44%) patients. During a mean of 43.44 ± 16.58 months of follow-up, case number 1 required a second intervention because of residual tumor, after 6 months from the curettage (Fig. 1). This residual tumor was treated successfully with radiofrequency treatment. Despite the rehabilitation protocol, 30° proximal interphalangeal joint flexion persisted in this patient. Case 2 has encountered a superficial infection which was treated with oral antibiotics. In cases 7 and 9, temporary superficial wound problems were encountered, which did not require treatment. No other complication was encountered (Table 2).

**Table 1:** Radiographic features of cases

Patient no.	X-ray	Tomography	MRI	SPECT-CT
1	Nidus + CT	Nidus + CT	CT + STE	HI
2	Non-specific	Non-specific	BME + STE	HI
3	Nidus + CT	Nidus + CT	BME	MI
4	Nidus + CT	Nidus + CT	STE	HI
5	Nidus + CT	Nidus + CT	Nidus + BME + STE	HI
6	Nidus + CT	Nidus + CT	BME	LI
7	Nidus + CT	Nidus + CT	STE + CT	MI
8	Non-specific	Nidus	Nidus	HI
9	CT	Sclerosis + CT	BME + CT	LI

*CT* cortical thickening, *STE* soft tissue edema, *BME* bone marrow edema, *HI* high intensity, *MI* moderate intensity, *LI* low intensity

**Table 2:** Demographics, clinical and pathological features of cases

Case no.	Age	Gender	Settlement	Delay^a^	Side	Treatment	Biopsy	Residual lesion	Complication	Follow-up^a^
1	26	K	Phalanx (proximal) (2nd)	9	Left	Burr-down	OO	+	PIP joint contracture	26
2	22	E	Metacarpal (2nd)	12	Left	Burr-down	OO	–	Superficial infection	72
3	15	E	Phalanx (middle) (2nd)	24	Right	Burr-down	OO	–	–	48
4	36	E	Phalanx (proximal) (5th)	6	Right	Burr-down	OO	–	–	56
5	31	E	Phalanx (proximal) (5th)	18	Right	Burr-down	OO	–	–	57
6	29	K	Phalanx (4th middle)	13	Left	Burr-down	OO	–	–	38
7	33	E	Phalanx (proximal) (4th)	9	Right	Burr-down	OO	–	Superficial wound problem	44
8	37	E	Phalanx (proximal) (2nd)	12	Left	Burr-down	OO	–	–	23
9	28	E	Phalanx (proximal) (5th)	16	Right	En-bloc	OO	–	Superficial wound problem	27

^a^Months

## Discussion

This study aimed to evaluate the epidemiology, radiologic features, surgical treatment options and functional outcomes. Male:female ratio was 3:1 in our series. Gender ratio in this present study adds a different ratio to the literature. But this different data may be related to small patient numbers. Variable male:female ratios were reported, but similarly with this present study there is a significant male predominance in literature [6, 9–11].

In our series, mean age was in the third decade. In the literature, OOs are usually seen below the fourth decade, reported in the second or third decade, and most patients are < 25 years old [9].

Phalanges were the most commonly affected bone and the most common involvement was in proximal phalanges. In addition, metacarpal bone settlement was the most rare one in our study, with a rate of 1/9 (11%). The most frequent location for OO in the hand region seems like proximal phalanges. Ozdemir et al. reported settlement as 11 (60%) proximal and 4 (20%) middle phalanges in 18 cases [11]. Jafari et al. reported settlement as 10 (40%) proximal, 5 (20%) distal phalanges and only 4/25 (16%) metacarpal in 25 cases [9].

Incidence of nidus formation was 6/9 (67%) on X-ray and mean time to diagnosis was 13.22 ± 5.44 months in this study. Similarly, Jafari et al. concluded that only 13 patients (52%) had the characteristic appearances of osteoid osteoma on X-ray, and they reported that the average time from the onset of symptom to successful treatment was 16.3 ± 11.1 months [9]. On X-rays, nidus formation may not be seen or may need to pass a long time to form [12]. Marcuzzi et al. reported that nidus incidence was 2/18 (11%) on X-ray. They concluded that the initial radiographs of almost all of the patients were normal. Also, they reported that the classical appearance of the disease can only be observed between 6 and 25 months. If the nidus has enough time to mature, it could be seen on X-ray [6]. However, we could not find additional information about their mean time to diagnosis. The duration of maturation and its pathogenesis are still unknown. Thus, initial X-ray examinations are often normal. This fact may be related to transposition, lack of periosteal reaction or cortical thickening. In the majority of our cases, nidus was detected on direct radiographs, but surely both planes should be taken. Typical appearance of nidus was mostly on anteroposterior and in lateral views only cortical thickening was noted. Also, normal scintigraphic findings may have been due to low metabolic activity in a mature osteoid osteoma [6].

Hand OO patients may be treated conservatively for extended periods. A large number of differentiating diseases directs the surgeon to shoot MR imaging instead of CT imaging. The diagnostic rate of CT imaging was high in our series. In CT imaging, cortical thickening obscured the nidus in only one patient. On the contrary, sclerosis may range from mild-cancellous to extensive-periosteal, and may obscure the nidus [13]. This result may be due to our use of thin section CT imaging in our cases. As a result, CT imaging reported as superior to X-ray and MR imaging in diagnosis, surgical planning and follow-up [9].

In MR imaging, nidus was detected in only 2 cases. The remaining cases had bone and/or soft tissue edema which obscured the nidus. MR imaging may depict the nidus and sclerosis because of adjacent bone marrow and soft tissue edema [14, 15]. Bone and/or soft tissue edema was seen on MR imaging, in all patients except one. Moreover, bone marrow and soft-tissue edema, joint effusion, and synovitis are better appreciated at MR imaging than at CT imaging [16]. Diffuse bone and/or soft tissue edema observed on MR imaging [17] may shift the diagnosis and long-term immobilization may be suggested. In our case series mean diagnostic time was similar to Jordan et al.’s systematic review [17]. The longest time to diagnose was 2 years due to nonspecific findings in X-ray and CT imaging. The reason for the delay was generalized edema due to pregnancy that hides the isolated finger edema. After the regression of postpartum edema, isolated finger swelling got attention. It should be noted that the prolongation of the treatment period causes social, economic, and psychological damage [18].

Pain was the most common symptom and all of our patients experienced night pain. In all patients, the pain was partially relieved with painkillers. In literature, the most common symptom is pain in hand OO [6, 9]. Pain severity increases at night and responds to prostaglandin inhibitors [19, 20]. Jafari et al. reported night pain rate as 21/25 (84%) and partial pain relief rate as 17/25 (68%); Marcuzzi et al. reported partial pain relief as 8/18 (44%) [6, 9]. Thus, our results regarding the night pain and pain relief are not compatible with the literature, and this shows that larger series are needed. The second common symptom has been reported as swelling [6, 9]. Swelling may be related to the rich vascular supply or permeability, results from the prostaglandins [21]. If OO settles near the joint, swelling and erythema misdirect the surgeon to a diagnosis of arthritis.

Many surgical techniques like en bloc resection, cortical peeling or burr-down with curettage, percutaneous curettage and alcoholization, laser coagulation, thermoregulation or radiofrequency ablation were defined [22–24]. En bloc resection requires bone grafting, and hand region is narrow for the percutaneous techniques [24, 25]. Cortical peeling is technically more difficult, especially with thick sclerosis, stripping of the cortex may not always be provided. Finding a cherry-red spot without disruption of typical appearance is possible with high-speed rolling burr. We performed the burr-down technique with curettage for all patients, except one. In one patient, 6 months after, a residual lesion was encountered which was treated successfully with radiofrequency. Because of a narrow surgical field, en bloc resection was performed only in one patient. We suggest burr-down method with high-speed burr and because of the low recurrence rate, grafts are not needed.

To the best of our knowledge, the most recent and most extensive series is from the year 2013 with a number of 25 cases [9]. Remaining papers are the mostly small number of case series or case reports. The rarity of hand OOs limits to report larger series. Retrospective, non-comparative manner and number of cases are the limitations, but the rarity obstructs the conditions for reporting a more extensive, prospective randomized-controlled series. There is still a need for more series to build more extensive reviews and evidence-based medicine.

## Conclusion

Osteoid osteoma usually seen below 25 years old, and rarely found over 40 years of age. There is male dominance with a male to female ratio of 3:1. Delay of diagnosis may be encountered because of many differential diagnoses. Local and sole presence of non-traumatic, prolonged swelling, pain responding to painkillers, tenderness, erythema, and sclerosis which is consistent with pain should remind the OO. When OO is suspected, CT imaging should be taken before the MR imaging. It should be kept in mind that the diagnostic value of thin-section CT imaging is higher than MR imaging. Because of superiority in soft tissue imaging, MR imaging should be an alternative tool in complex cases. Unroofing and curettage with “Burr-down” method seems to be effective in preventing residual tumors or relapses.
